# NLRP3 inflammasome expression in pediatric asthma: sputum-based insights, inflammatory mechanisms, and targeted therapeutic strategies

**DOI:** 10.1186/s13223-025-01001-1

**Published:** 2025-11-29

**Authors:** Mohammed AbuBaha, Samia Aldwaik, Bara Abubaha, Anwar Zahran, Dana Sandouka, Kareem Istetieh, Husam Hamshary, Mohammad Abushehadeh, Sarah Saife, Nadeen Sandoqah

**Affiliations:** Department of Medicine, An-Najah National University, Nablus, Palestine

**Keywords:** Asthma, Pediatric, NLRP3, Inflammasome, Immune, Inflammatory, Pediatric asthma

## Abstract

Asthma is a common chronic inflammatory disease in children. Pediatric asthma has a wide range of immunologic phenotypes and different treatment responses. Recent data from various studies suggest that the NOD-like receptor family pyrin domain-containing 3 (NLRP3) inflammasome, a cytosolic multiprotein complex, has a central role in innate immunity, may be implicated in pediatric asthma pathogenesis, especially in the neutrophilic corticosteroid-resistant type. This review article investigates the mechanism of NLRP3 inflammasome activation as well as its role in airway inflammation and its expression in pediatric asthma based on sputum studies. The findings emphasize an association between elevated NLRP3 levels and poor asthma control. The need for novel treatments is important because Current using therapies such as corticosteroids and biologics demonstrate variable efficacy. According to data, their efficacy heavily depends on the underlying inflammatory phenotype. Biologics such as mepolizumab, benralizumab, and dupilumab are well known for their corticosteroid-sparing effects especially in cases of severe eosinophilic asthma. even though, their therapeutic benefits are limited when it comes to neutrophilic or steroid-resistant phenotypes. NLRP3 inhibitors are new, promising treatments which emerged recently and show potential capability in reducing airway inflammation in animal models. Furthermore, NLRP3-driven inflammation appears to play a role not only in asthma but also in inflammatory bowel disease and juvenile idiopathic arthritis, indicating a wider relevance for therapies that target the inflammasome pathway. Although promising data, application of this data in clinical practice is still challenging due to many causes, including diagnostic challenges, ethical considerations in trials involving children, and the lack of approved NLRP3 inhibitors for use in children. More research is required and essential to confirm that NLRP3 could be used as a biomarker or therapeutic target in pediatric asthma.

**Graphical abstract:**

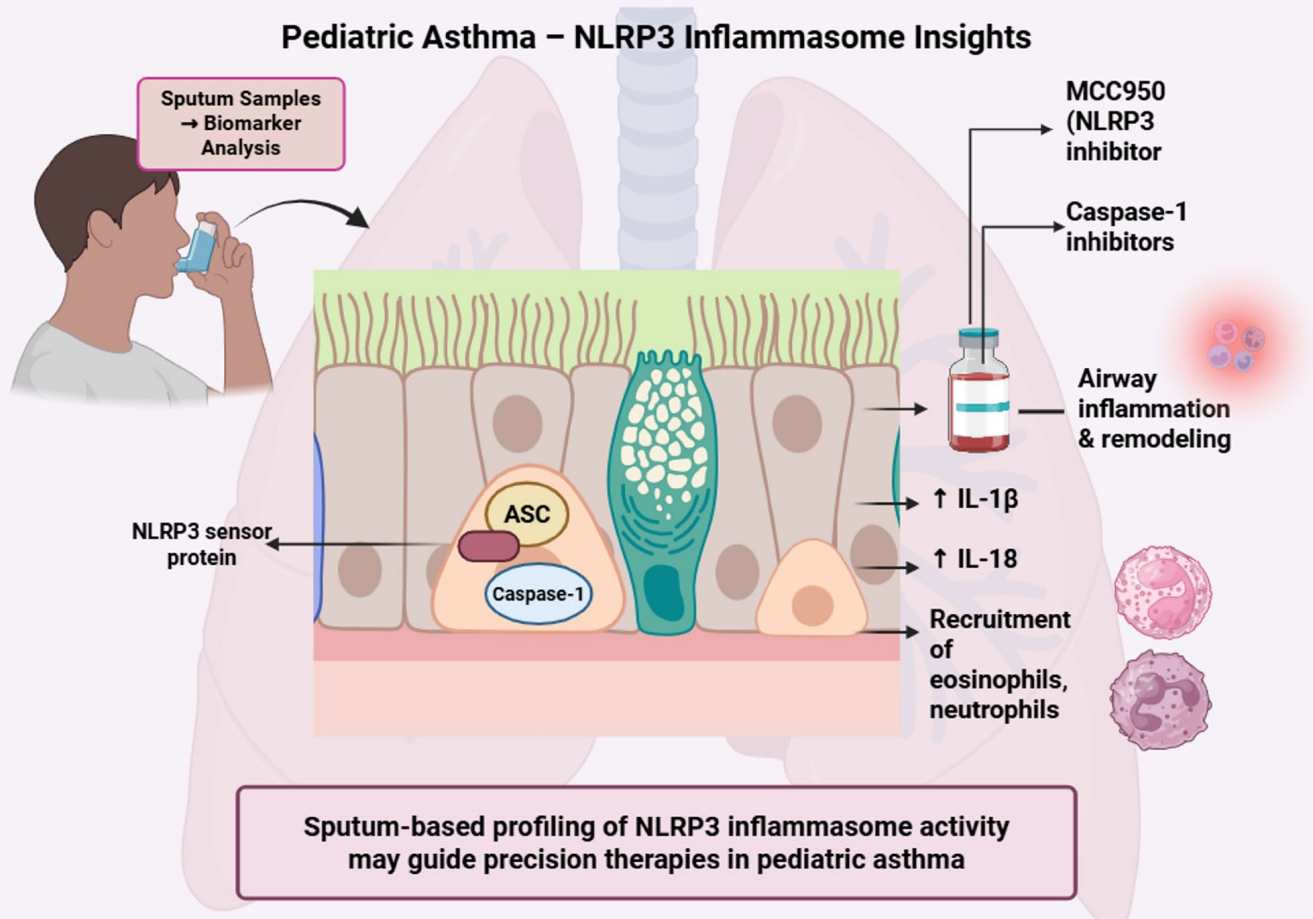

## Introduction

Asthma is a chronic, long-term inflammatory disease that causes narrowing of the airways. It has a high prevalence in children. Asthma significantly impairs quality of life and increases healthcare utilization worldwide; it is also associated with increased morbidity [1]. The risk of long-term pulmonary complications is linked to early onset and frequent exacerbations of pediatric asthma [1].

Pathophysiology of asthma involves airway inflammation, which manifests in children as a spectrum of immunologic phenotypes. The first type, which is an eosinophilic inflammation, is usually associated with type 2 immune responses; it is very common and often considered to be corticosteroid-responsive [1, 2]. However, a second type, which is neutrophilic or mixed granulocytic airway inflammation, is usually associated with more severe disease, increased risk of exacerbation, and reduced responsiveness to standard therapies. The underlying mechanism of these non-eosinophilic phenotypes remains poorly understood [1, 2].

The NOD-like receptor family pyrin domain-containing 3 (NLRP3) inflammasome is a cytosolic multiprotein complex; its main function is to detect pathogen-associated molecular patterns (PAMPs) and danger-associated molecular patterns (DAMPs) [3–6]. Activation of NLRP3 involves oligomerization with Apoptosis-Associated Speck-Like Protein (ASC) and pro-caspase-1, and this will lead to activation of the caspase-1 molecule, and the result of this process is the secretion of interleukin-1β (IL-1β), interleukin-18 (IL-18), and gasdermin D-mediated pyroptotic cell death [2–11]. This pathway, which is critical in innate immunity, has been linked to the pathogenesis of a wide range of inflammatory and autoimmune diseases [1–12].

According to recently published data, which suggests that activation of NLRP3 inflammasome may influence asthma severity, phenotype heterogeneity, and treatment resistance, especially in neutrophilic or corticosteroid-resistant pediatric asthma [1, 2, 7, 11, 12]. Targeting NLRP3-driven cytokine signaling in specific pediatric asthma is rational because sputum-based studies and translational models have started to clarify the pathway’s function in airway inflammation and remodeling [1, 2, 7, 11, 12]. Understanding of the relationship between NLRP3 inflammasome activity and pediatric asthma pathobiology may help in the development of novel, targeted therapeutic strategies for children [1, 2, 7, 11, 12].

## Biology of the NLRP3 inflammasome

Understanding the NLRP3 structure and activation mechanisms is essential, as dysregulation of this complex has been increasingly implicated in the pathogenesis and heterogeneity of pediatric asthma. This section will explore how the NLRP3 inflammasome functions at the molecular level, linking innate immune sensing to airway inflammation and remodeling relevant to asthma phenotypes and severity.

### Structure and activation cascade

The NLRP3 inflammasome is a big, cytosolic multiprotein complex that is a critical part of the innate immune system that detects cellular stress and infection. The NLRP3 protein has three main parts: an N-terminal pyrin domain (PYD) that helps proteins interact, a central NACHT domain that controls ATPase activity and oligomerization, and a C-terminal leucine-rich repeat (LRR) domain that helps the protein regulate itself and sense ligands. When not in use, NLRP3 is mostly in an autoinhibited monomeric or dimeric state. When NLRP3 is activated, it changes shape so that it can oligomerize, creating a structure that looks like a disk or cage [13]. Recent investigations using cryo-electron microscopy (cryo-EM) have shown that NIMA-related kinase 7 (NEK7), a serine/threonine kinase that directly binds to the LRR domain of NLRP3, is also a member of the active inflammasome complex. This binding keeps NLRP3 in its active shape and helps it come together to create bigger structures [14].

The NLRP3 inflammasome is turned on in two very controlled processes. PAMPs or cytokines like lipopolysaccharides (LPS) or tumor necrosis factor (TNF) start the first phase, called priming. These molecules trigger NF-κB signaling. This process makes it easier for NLRP3 and pro-IL-1β to be transcribed and changes NLRP3 after it has been translated, which “licenses” it for later activation [15]. Potassium efflux, mitochondrial malfunction, and reactive oxygen species (ROS) are just a few of the things that can start the second step of activation. It is well known that potassium efflux is a universal signal that activates NLRP3. NEK7 works after K⁺ leaves the cell to help put together the inflammasome. When it is turned on, NLRP3 oligomerizes and brings in the adaptor protein ASC (apoptosis-associated speck-like protein containing a CARD) through PYD-PYD interactions. After that, ASC polymerizes into filamentous structures, creating a big cytosolic speck that serves as a scaffold for the assembly of pro-caspase-1 through CARD-CARD interactions [16–18]. This assembly lets caspase-1, a cysteine protease that is very important for downstream effector actions, cut itself and become active.

Caspase-1 changes the precursor cytokines pro-IL-1β and pro-IL-18 into their mature, secretory versions. IL-1β is a powerful mediator that brings neutrophils to the site of inflammation and makes it worse. IL-18, on the other hand, boosts the synthesis of interferon-γ, which further activates the immune system. At the same time, caspase-1 cuts gasdermin D (GSDMD), freeing its N-terminal fragment, which subsequently oligomerizes and becomes part of the plasma membrane, making holes. Pyroptosis is a type of programmed cell death that causes cells to expand, their membranes to break, and their cytosolic contents to be released. Recent research shows that IL-1β and IL-18 can be released without fully breaking down cells [19]. This means that the release of cytokines and pyroptosis may not happen at the same time for every type of cell and stimulation. GSDMD-dependent pores make it easier for cytokines to be released. Their creation is affected by other things, like the ubiquitination of the GSDMD N-terminal domain, which changes how pores form and where they are located on the membrane [20].

### Triggers of NLRP3 activation

**Fig. 1:**
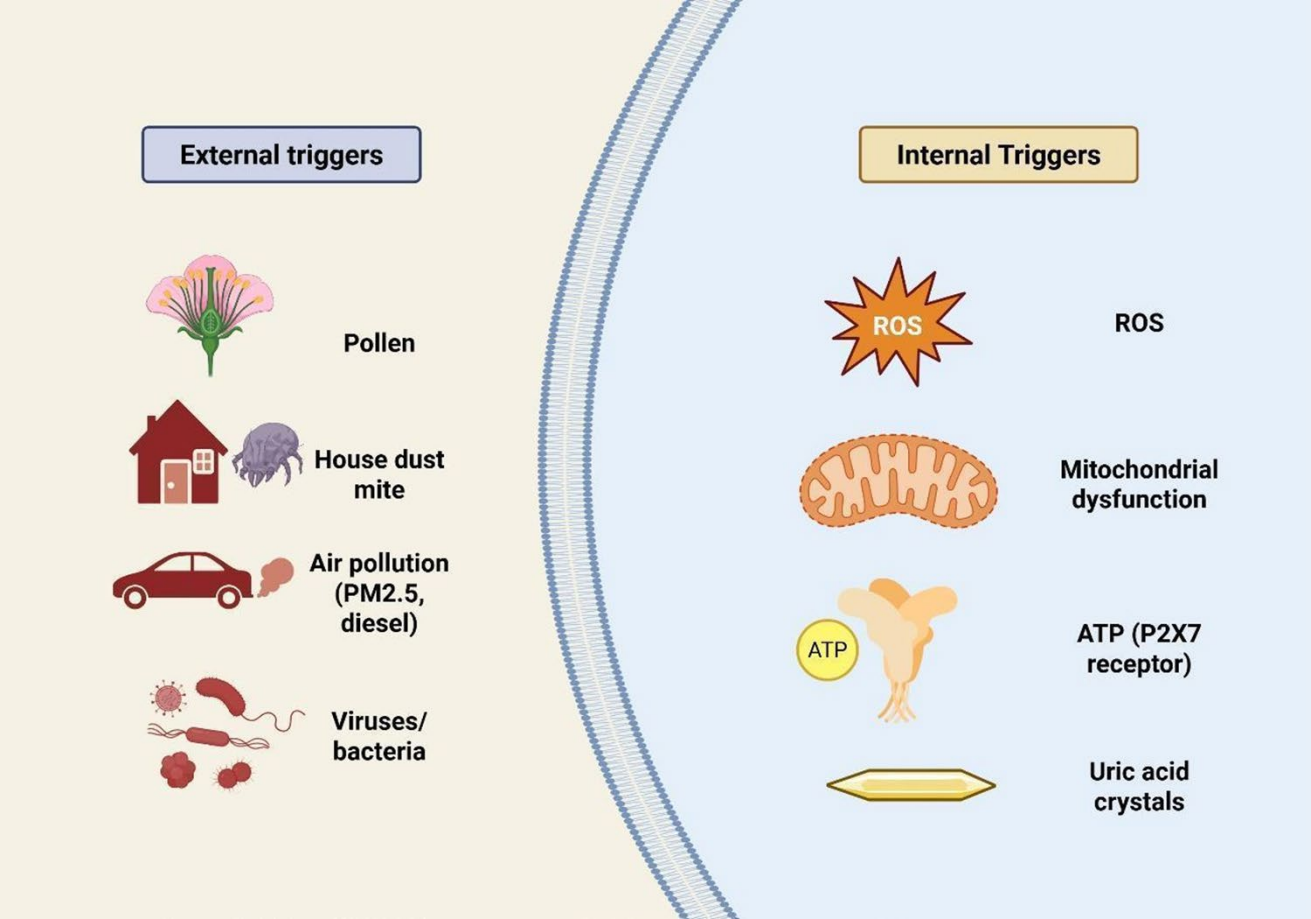
Triggers of NLRP3 Inflammasome Activation in Pediatric Asthma. This illustration summarizes the main exogenous and endogenous triggers that activate the NLRP3 inflammasome in pediatric asthma. External stimuli such as allergens (e.g., pollen, dust mites), pollutants (e.g., PM2.5, ozone), and respiratory pathogens induce oxidative stress and epithelial injury. Concurrently, internal danger signals—like extracellular ATP, mitochondrial ROS, and uric acid crystals—further promote NLRP3 assembly and activation. Together, these triggers initiate a cascade that amplifies airway inflammation through the release of IL-1β and IL-18, contributing to asthma severity and steroid resistance. PM2.5: Particulate matter 2.5, ROS: Reactive oxygen species, ATP: Adenosine triphosphate, P2X7: P2X purinoceptor 7

The NLRP3 inflammasome can be activated by various external factors in the airway, including allergens, pollutants, and respiratory pathogens (Fig. 1). Common triggers such as pollen and house dust mite proteins can induce oxidative stress in airway epithelial cells, leading to the production of ROS [21, 22]. This excess of ROS activates the NLRP3 inflammasome, resulting in the release of pro-inflammatory cytokines like IL-1β and IL-18. These cytokines recruit immune cells to the site of inflammation, exacerbating conditions in the airways, which are critical steps in the development of asthma and other allergic airway diseases [23]. Environmental pollutants, including particulate matter (PM2.5), ozone, and diesel exhaust particles, can significantly activate the NLRP3 inflammasome. These pollutants compromise the integrity of epithelial cells and promote ROS production, which acts as a secondary messenger to activate the inflammasome [24, 25]. Experimental models demonstrate that exposure to these pollutants intensifies airway inflammation and hyperresponsiveness, with these effects being notably reduced in the absence of NLRP3 [26, 27]

Additionally, respiratory infections caused by bacteria, viruses, or fungi represent another significant category of NLRP3 activators. Host pattern recognition receptors identify PAMPs from these microbes, leading to mitochondrial dysfunction, ionic imbalances, and the production of ROS. These simultaneous events facilitate the assembly and activation of the NLRP3 inflammasome. While this process aids the body in combating infection, it can also result in tissue damage if not properly regulated. In addition to exogenous triggers, several endogenous signals are integral to NLRP3 activation in the airway microenvironment [26]. Extracellular ATP, released from damaged or stressed airway cells, is a well-characterized activator of the NLRP3 inflammasome. ATP binds to the purinergic P2X7 receptor on immune cells, inducing potassium efflux, a critical upstream event required for inflammasome assembly and activation [26, 28].

ROS, generated as a byproduct of mitochondrial dysfunction or environmental insult, act as essential secondary messengers in the NLRP3 activation cascade. Elevated ROS levels facilitate the oligomerization of NLRP3 and enhance downstream signaling, directly linking oxidative stress to inflammasome-mediated inflammation. Uric acid crystals represent another potent endogenous trigger. In the context of airway inflammation, uric acid can accumulate as a result of increased cell turnover or tissue injury. When phagocytosed by immune cells, these crystals cause lysosomal destabilization and further mitochondrial ROS production, both of which are necessary for NLRP3 activation. Notably, soluble uric acid has also been implicated in inflammasome activation through redox-dependent mechanisms, underscoring the complexity of endogenous danger signaling in the airways [29].

These results show that NLRP3 inflammasome activation in the airways is caused by many different things, including environmental exposures, infectious agents, and stressors that come from within the body. These factors work together to cause inflammatory responses that are key to the development of airway diseases. (Table 1).

**Table 1:** Common environmental triggers of NLRP3 activation and their inflammatory consequences

Environmental Trigger	Mechanism of NLRP3 Activation	Inflammatory Outcome	Reference
Pollen, HDM	ROS generation in epithelial cells	IL-1β, IL-18 secretion, airway inflammation	Theofani et al., 2019; Bauer et al., 2024
PM2.5, Ozone	Epithelial ROS production, mitochondrial dysfunction	NLRP3 activation, airway remodeling	Caceres et al., 2024; Xiong et al., 2021
Respiratory pathogens	Mitochondrial damage, ATP release	Neutrophil recruitment, tissue injury	Chen et al., 2022; Wang et al., 2025

NLRP3: NOD-like receptor family pyrin domain-containing 3, HDM: House dust mite, ROS: Reactive oxygen species, IL: Interleukin, PM2.5: Particulate matter 2.5, ATP: Adenosine triphosphate

### NLRP3 and airway inflammation

A lot of studies, both on animals and on individuals with asthma, show that the NLRP3 inflammasome is a big part of airway inflammation [30, 31]. When NLRP3 is removed or blocked in mice with allergic airway illness, the airways become less inflamed, fewer eosinophils get into them, and less mucus is generated after the animals are exposed to allergens. For example, mice that do not have NLRP3 or its downstream effectors, such as ASC or caspase-1, have considerably lower levels of airway hyperresponsiveness and inflammatory cytokines after being exposed to house dust mite (HDM) or ovalbumen (OVA) than normal mice [32]. These data reveal how crucial NLRP3 is for modulating the immune responses that induce allergic airway inflammation.

In adults with asthma, airway tissues and immune cells from patients, especially those with severe or steroid-resistant phenotypes, have been reported to have larger amounts of the NLRP3 inflammasome and to be more active. People with asthma had more NLRP3, ASC, caspase-1, and the pro-inflammatory cytokines IL-1β and IL-18 in their bronchoalveolar lavage fluid and sputum samples. These levels depend on how bad the disease is and how the airways alter shape [22, 33]. Giving NLRP3 inhibitors to animals lowers airway hyperresponsiveness and inflammation, which supports the concept that targeting this pathway in human asthma is helpful [33]. Researchers believe that long-term activation of the NLRP3 inflammasome creates a feedback loop that keeps airway inflammation running and makes it worse. NLRP3 is turned on when you are around irritants, allergens, or infectious agents all the time. This makes IL-1 and IL-18. Not only do these bring in and activate more immune cells, but they also generate more endogenous danger signals like ATP and ROS. These signals make NLRP3 even more active.

This loop of feed-forward can also cause abnormalities in the airways, make too much mucus, and cause kinds of asthma that do not respond to steroids [29]. Long-term activation of NLRP3 in animal models is connected to higher levels of helper type 2 cytokines and helper type 17 cytokines, higher levels of serum amyloid A, and more damage to tissues. All of these things are symptoms of severe and long-lasting asthma. Also, research suggests that NLRP3 causes pyroptosis in airway macrophages and epithelial cells, which makes tissue damage worse and keeps the inflammatory environment going [34, 35]. These data show that NLRP3 plays a role in both starting and keeping airway inflammation going. This makes it a very important target for treating asthma and other airway illnesses.

## NLRP3 expression in pediatric asthma

This section explores the detection and measurement of NLRP3 inflammasome components in sputum samples from pediatric asthma patients. It examines how analyzing sputum provides important insights into the degree of airway inflammation, the relationship between NLRP3 expression and asthma severity, and its potential as a biomarker for monitoring disease progression and response to therapy in children.

### Detection in sputum samples

Induced sputum analysis is a recognized, noninvasive method for assessing airway inflammation in children with asthma. By inhaling hypertonic saline, patients produce sputum that can be analyzed for inflammatory cells and soluble mediators, providing important information on disease phenotypes and treatment response. This approach is particularly valuable in pediatrics, as it avoids invasive procedures such as bronchoscopy while allowing repeated, longitudinal sampling of airway biomarkers [36, 37].

Basic precautions are important to ensure reliable sampling and patient safety. Patients are instructed to rinse their mouths beforehand to minimize contamination, and a specimen of at least 1–2 mL is usually considered adequate. The procedure should be discontinued if significant adverse symptoms such as chest tightness, wheezing, or dyspnea occur. Pretreatment with a short‑acting bronchodilator and monitoring of lung function are recommended to reduce the risk of bronchoconstriction, and sputum induction is best conducted under medical supervision [36, 38].

Recent studies highlight the utility of this method in biomarker discovery and phenotyping. A 2024 cross‑sectional study demonstrated significantly elevated levels of NLRP3, IL‑1β, and IL‑6 in children with asthma compared to healthy controls, with the highest levels observed in those with moderate to severe disease. Increased NLRP3 correlated negatively with lung function indices (FEV_1_/FVC, PEF, FVC predicted) and was associated with airway inflammation, supporting its promise as a marker of disease severity. Additional findings implicate inflammasome‑related cytokines (IL‑1β, IL‑18) in neutrophilic asthma phenotypes, poor asthma control, and corticosteroid resistance in pediatric disease. Collectively, these results underline the value of induced sputum analysis both as a research tool and as a potential contributor to phenotype‑driven clinical management of childhood asthma [30].

The expression of NLRP3 and IL‑1β in sputum has been associated with neutrophilic asthma phenotypes in children, correlating with greater disease severity, poor asthma control, and corticosteroid resistance [39]. These findings emphasize the role of the NLRP3 inflammasome and its downstream cytokines (IL‑1β, IL‑18) in the development and clinical manifestation of severe childhood asthma. By promoting eosinophil recruitment and triggering mast cell degranulation, these mediators contribute to the progression of allergic inflammatory processes [23, 40]. (Table 2).

**Table 2:** Biomarkers from Induced Sputum and Their Association with Asthma Severity

Biomarker	Source	Correlation with Asthma Severity	Additional Notes	Reference
NLRP3	Induced sputum	Positive	Higher levels in moderate/severe cases	Li and Liu, 2024
IL-1β	Induced sputum	Positive	Linked to neutrophilic phenotype	Li and Liu, 2024; Simpson et al., 2014
IL-6	Induced sputum	Positive	Correlated with NLRP3 expression	Li and Liu, 2024
FEV1/FVC	Lung function test	Negative	Inversely related to NLRP3 levels	Li and Liu, 2024

NLRP3: NOD-like receptor family pyrin domain-containing 3, IL: Interleukin, FEV1: Forced expiratory volume in 1 s, FVC: Forced vital capacity

### Clinical and inflammatory phenotypes

**Fig. 2:**
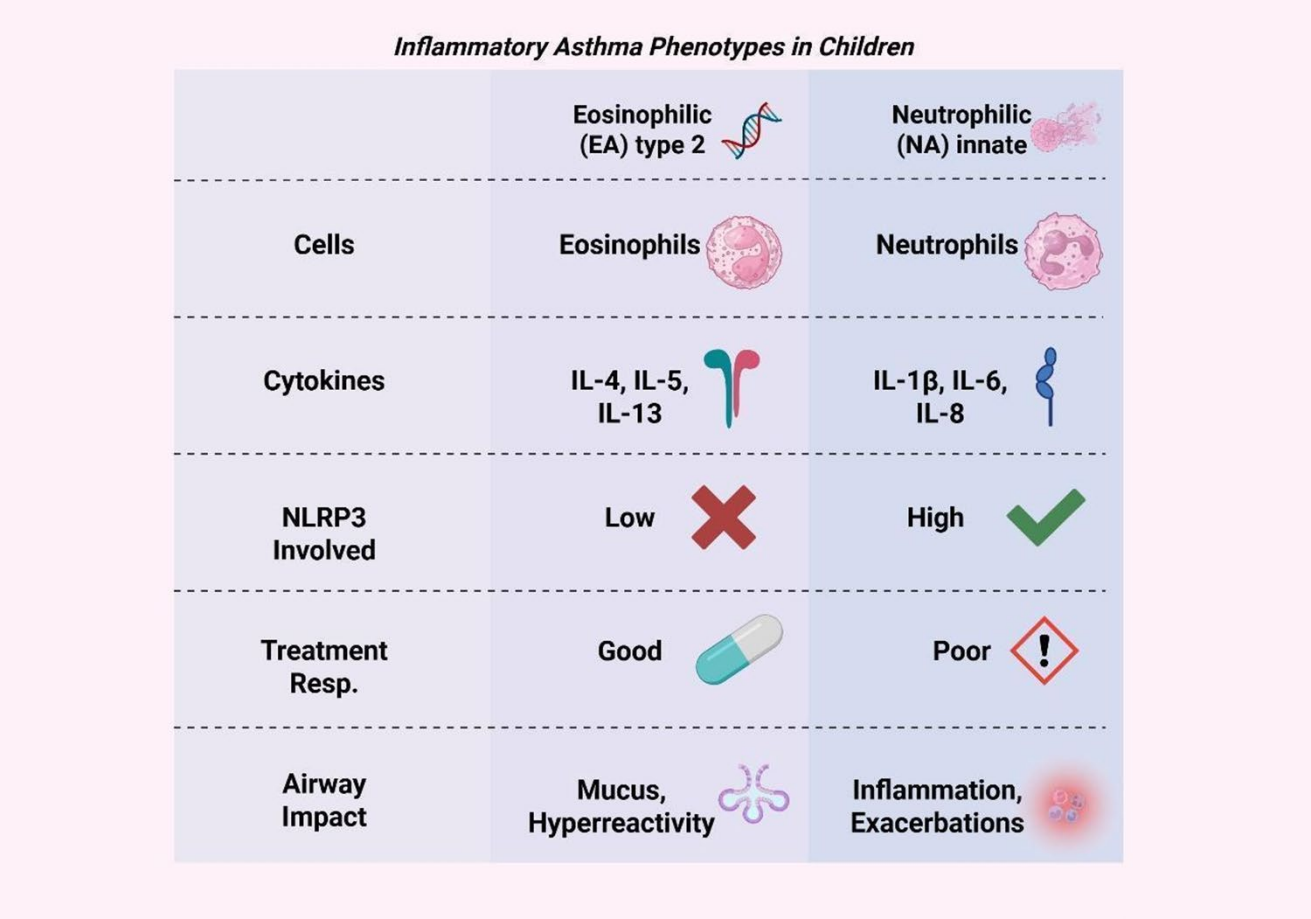
Inflammatory asthma phenotypes in children. Comparison between eosinophilic (type 2) and neutrophilic (innate) asthma phenotypes, highlighting differences in cytokine profiles, NLRP3 involvement, treatment response, and airway impact. IL: Interleukin, NLRP3: NOD-like receptor family pyrin domain-containing 3, Resp.: Response

Asthma is a chronic inflammatory disease of the lower airways with different overlapping types. It can be broadly divided into type 2-high (type 2) (T2) form and type 2-low (non-type 2) (non-T2) (Fig. 2). T2 asthma is linked to atopy and eosinophilic inflammation, which involves cytokines such as IL-4, IL-5, IL-9, and IL-13. Those activate IgE production, thus eosinophilic recruitment, airways hyperresponsiveness, and mucus overproduction. Early-onset allergic asthma, adult-onset eosinophilic asthma, and exercise-induced bronchoconstriction fall under this subtype. Neutrophilic asthma related to smoking or post-viral inflammation, and obesity-associated asthma, which are associated with T helper type 17 and type 1 cytokines such as interferon γ, TNFα, and IL-17. Regardless of type, repeated epithelial injury can lead to chronic inflammation and airway remodeling via epithelial-mesenchymal transition (EMT), resulting in changes like smooth muscle hypertrophy, angiogenesis, and barrier dysfunction [41].

In pediatric asthma, eosinophilic asthma (EA) and neutrophilic asthma (NA) represent two major inflammatory phenotypes with distinct clinical and biological characteristics. EA in children is typically marked by elevated eosinophils in sputum and blood. It is often associated with type 2 (T2) inflammation and generally responds well to inhaled corticosteroids and targeted biologic therapies. In contrast, NA is defined by increased neutrophils in sputum and higher levels of neutrophil-associated cytokines such as IL-1β and IL-8 and is more frequently linked to severe disease, poorer asthma control, and reduced responsiveness to corticosteroids. Pediatric NA is also associated with greater airflow limitation and more frequent exacerbations and may be influenced by factors such as respiratory infections and environmental exposures [23].

Research has shown the differences in pathophysiological mechanisms. With EA, it is primarily driven by Type 2 cytokines and eosinophil activation. However, NA involves innate immune responses, inflammasome activation like NLRP3, and neutrophil extracellular trap formation. Recognizing these differences and the complexity within each phenotype plays a crucial part in accurate diagnosis, leading to personalized therapy and improving outcomes for children with asthma [23, 42].

### Challenges in pediatrics airway sampling

Accurate assessment of NLRP3 inflammasome expression in children with asthma hinges on obtaining reliable airway samples. Induced sputum is the standard medium for measuring inflammasome activity and cytokine profiles [30]. One of the biggest challenges is getting high-quality airway samples from pediatric groups. Accurate inflammatory phenotyping may be hampered by the limited success rates, technical difficulties, and frequent contamination from upper airway secretions associated with induced sputum sampling. Up to 29% of pediatric sputum samples may not be of adequate quality, according to a recent cross-sectional study [43]. This underscores the necessity of strict quality control to precisely identify disease-microbiota relationships. To overcome these constraints, new noninvasive instruments are being created. For example, exhaled breath condensate (EBC), an approach that is accomplished by cooling exhaled air, which contains aerosolized particles and volatile substances from the breath, helps in assessing local airway inflammation in pediatric asthma patients [44]. By making it easier to detect cytokines such as IL-13 and IL-17A. However, more standardization and validation are needed before EBC is widely used in clinical practice [44, 45].

Gene expression profiling with nose swabs is one method that appears promising for getting around these limitations [46, 47]. A 2025 multicenter study showed that nasal epithelial sampling is a useful and less invasive method of identifying children’s asthma endotypes than sputum induction. Particularly in populations with a significant burden of asthma, this approach may increase the accuracy of phenotyping and tailored treatment [48]. This less-invasive approach could greatly enhance phenotyping accuracy in pediatric NLRP3-related asthma studies when sputum collection proves difficult.

## Mechanistic implications in asthma pathogenesis

When activation of the NLRP3 inflammasome occurs, the pro-inflammatory cytokines IL-1β and IL-18 are released and processed by caspase-1, a crucial element in airway remodeling in asthmatic children. IL 1β has been shown to enhance the pathophysiological features of asthma, such as epithelial injury, goblet cell proliferation, excessive mucus production, and airway smooth muscle enlargement. In steroid-resistant asthma models, Kim et al. recently demonstrated that higher levels of NLRP3, caspase 1, and IL-1β exacerbate neutrophil-driven inflammation and airway remodeling [49]. At the same time, IL 18 enhances the activity of matrix metalloproteinases (such as MMP 1, 2, 9), which facilitates the remodeling of the extracellular matrix and increases the thickness of the airway wall while directly stimulating epithelial proliferation, mucus metaplasia, and contraction of smooth muscle; mice lacking IL 18 exhibit significantly reduced airway remodeling in OVA-challenged models [50].

In addition to structural impacts, IL 1β and IL 18 amplify inflammatory immune pathways, particularly promoting helper type 17 differentiation alongside IL 23, and encouraging IL 17A-mediated neutrophil recruitment—a characteristic of severe and steroid-resistant asthma [51]. Increased sputum IL-1β is closely associated with neutrophilic inflammation and higher levels of neutrophil extracellular traps (NETs) in children, supporting the pathogenic significance of this pathway [49].

The NLRP3 inflammasome further influences essential interactions with additional immune cells. When exposed to allergens like house dust mite (HDM) or Der p1, epithelial cells, macrophages, and dendritic cells trigger NLRP3, resulting in the release of IL-1β. This enhances the maturation of dendritic cells and biases T cell polarization towards helper type 2 and helper type 17 types [52]. In vitro studies additionally demonstrate that IL-1β from macrophages increases eosinophil recruitment and airway inflammation [49]. Notably, in the context of asthma exacerbations caused by rhinovirus, IL 1β and IL 18 play crucial roles in maintaining immune equilibrium: a lack of IL 1β leads to diminished helper type 17 responses and reduced mucin gene expression (IL 25, IL 33), promoting a helper type 2 shift, whereas IL 18 has been found to alter ILC2s to produce IL 17, thereby strengthening pathogenic mixed-type inflammation [52].

Environmental factors, like air pollutants (PM_2.5_/PM_10_), viral infections (especially rhinovirus), and allergens, act as strong triggers for the NLRP3 inflammasome. Airborne particulate matter stimulates ROS generation and destabilizes lysosomes in epithelial and immune cells, resulting in NLRP3 activation and IL-1β release, causing disruption of the epithelial barrier, infiltration of neutrophils, and increased α-SMA and collagen accumulation in the airway wall [53]. Rhinovirus functions by activating caspase 1 via TLR2, leading to increased secretion of IL-1β and IL-18, which results in heightened inflammation and reduced steroid effectiveness [54]. Extra allergens like HDM further activate epithelial inflammasomes via ROS-related pathways, intensifying airway inflammation [53].

Finally, genetic tendencies similarly affect inflammasome function. A recent study involving a pediatric cohort found that NLRP3 polymorphisms (such as rs10925023, rs10754558), along with variants in MAVS and IL-18, were significantly linked to a higher risk of asthma, increased IgE levels, and greater disease severity [55].

## Therapeutic targeting of NLRP3 in pediatric asthma

**Fig. 3:**
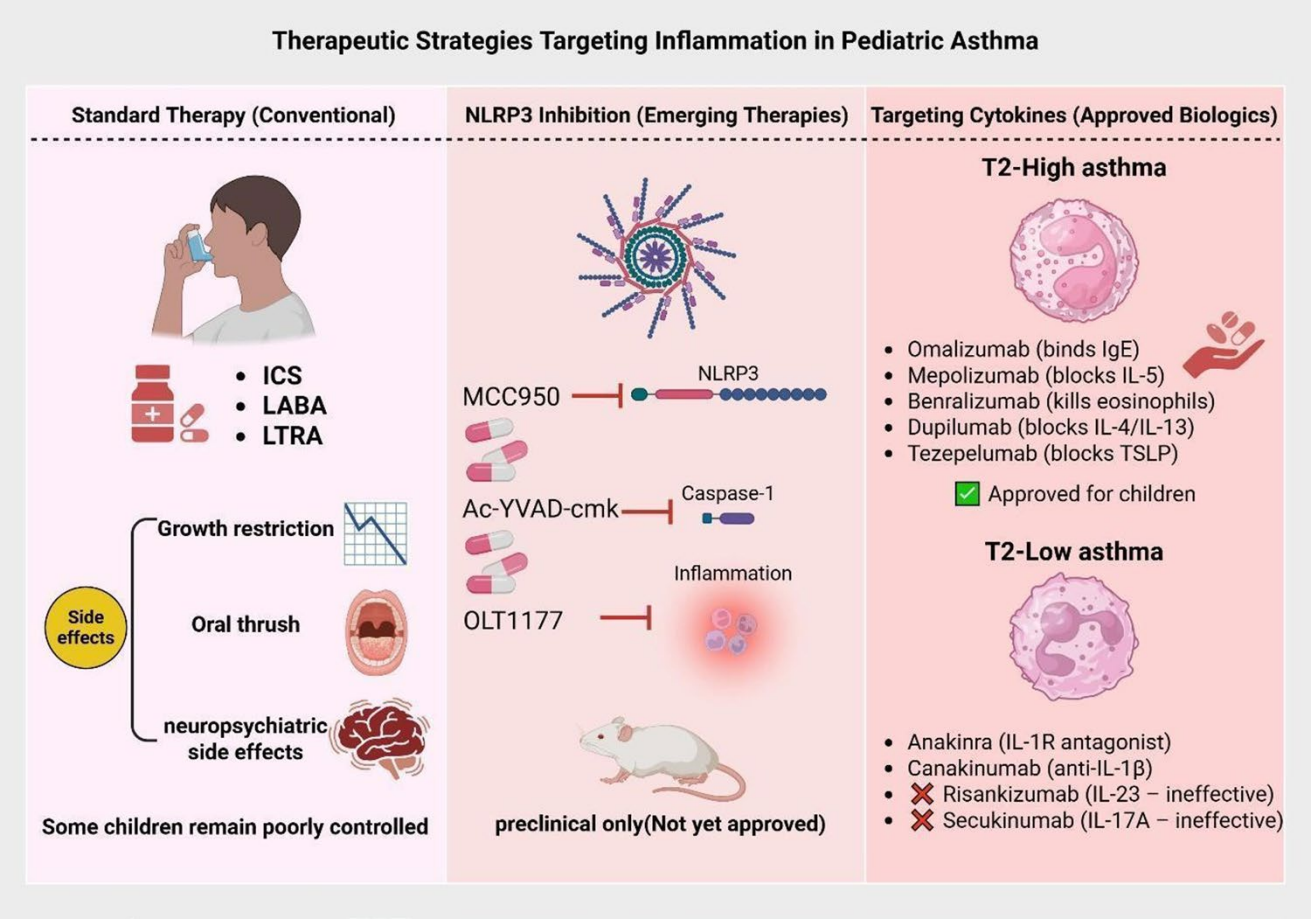
*Therapeutic strategies for pediatric asthma, with emphasis on NLRP3-targeted approaches.* This schematic illustrates three major categories of asthma therapy: conventional anti-inflammatory treatments (e.g., inhaled corticosteroids, LABA, and LTRA), emerging experimental strategies targeting the NLRP3 inflammasome pathway (including MCC950, Ac-YVAD-cmk, and OLT1177), and approved biologic therapies aimed at downstream cytokines involved in T2-high and T2-low asthma phenotypes. While conventional treatments are widely used, their limitations in steroid-resistant or neutrophilic asthma highlight the need for novel approaches. Experimental NLRP3 inhibitors and cytokine-blocking agents represent potential therapeutic advancements, especially for difficult-to-treat pediatric asthma. ICS: Inhaled corticosteroids, LABA: Long-acting beta-agonist, LTRA: Leukotriene receptor antagonist, NLRP3: NOD-like receptor family pyrin domain-containing 3, Ac-YVAD-cmk: Acetyl-YVAD-chloromethylketone, Ig: immunoglobulin, IL: Interleukin, TSLP: Thymic stromal lymphopoietin

In pediatric asthma, the goal of treatment is to prevent chronic symptoms, maintain lung function, and allow for normal daily activity, thus keeping children “symptom-free” [56]. Every asthmatic child is recommended to avoid triggers and allergens identified by history or skin-prick test, or serum-specific IgE testing to reduce symptoms, and this should be addressed in each visit [56]. Determining the initial treatment of pediatric asthma requires assessment of the severity of symptoms and the likelihood of future exacerbations. In infants and young children, regimens that contain glucocorticoids are preferred as initial therapy [57]. An overview of conventional, biological, and emerging NLR3-targeted treatment approaches in pediatric asthma are highlighted below (Fig. 3).

### Anti-inflammatory treatments and limitations

Global Initiative for Asthma (GINA) 2025 guidelines recommend a stepwise approach for treatment: Step 1 and 2 recommend using as-needed low dose ICS-formoterol with or without SABA (SABA-only is no longer recommended as using ICS-formoterol has shown to decrease exacerbations significantly), if remains poorly controlled then ensure correct usage of regiment and correct avoidance of exposure, if still fail then step 3 is starting maintenance and reliever therapy (MART) of ICS-formoterol on top of as needed ICS-formoterol, step 4 involves increasing the dose to medium intensity, step 5 involves referral for phenotyping, possible biologic treatments, and additional controllers such as long-acting muscarinic antagonists (LAMAs) [58]. Young children (< 5 years) may require nebulizers over inhalers due to difficulty using inhalers [59]. Although these anti-inflammatory treatments are first-line, they are notorious for having harmful effects, especially on children. LABA monotherapy is known to increase the risk of severe asthma exacerbations (SAEs) and asthma-related death [60]. ICS monotherapy can cause local side effects like cough, oral thrush, dysphonia, and hoarseness of voice [61], and systemic side effects like growth restriction, especially in higher doses [60], and a minor decrease in post-ACTH stimulation cortisol values, though it had no clinical value [62]. LTRAs were shown to be less effective regarding the reduction of airway inflammation and remodeling compared to ICS [63] and increased the risk of neuropsychiatric symptoms and sleep disorders in children [64]. Another limitation of treating pediatric asthma is severe therapy-resistant asthma (STRA) in a minor group of children (2–5% of all asthmatic children), which is challenging to treat [65] and uncontrolled pediatric asthma despite adherence to the treatment regimen and correct inhaler techniques [66]. These limitations warrant and necessitate the search for new and more asthma-specific substitutes, especially for neutrophilic (non-allergic) subtypes [26].

### NLRP inhibitors

**Fig. 4:**
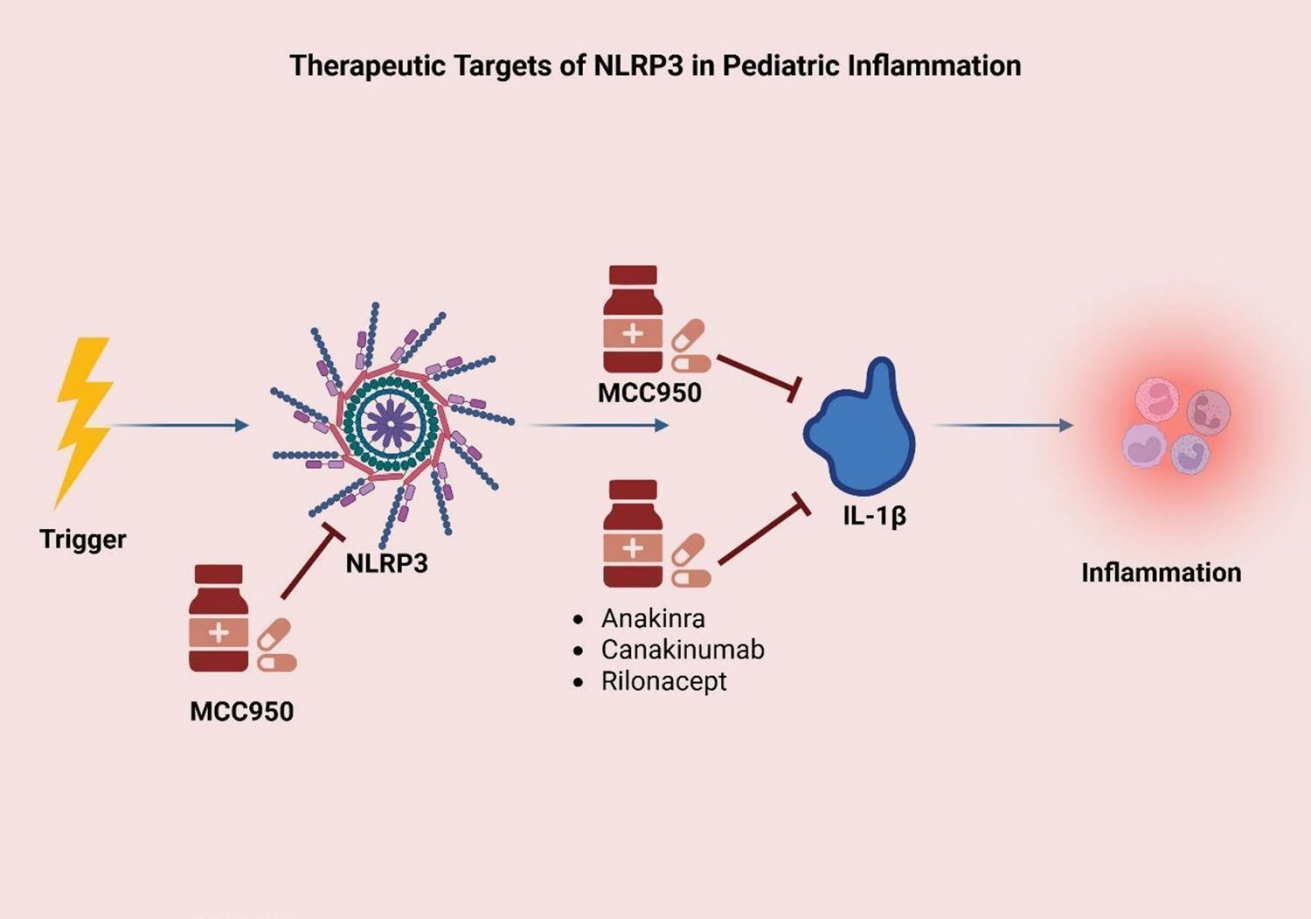
Therapeutic Targeting of the NLRP3 Inflammasome Pathway in Pediatric Inflammation. This diagram illustrates key molecular targets in the NLRP3 inflammasome pathway relevant to pediatric inflammatory diseases, including asthma. Environmental or endogenous triggers activate the NLRP3 complex, leading to IL-1β release and downstream inflammation. Specific inhibitors such as MCC950 target the inflammasome directly, while agents like Anakinra, Canakinumab, and Rilonacept block IL-1β signaling, reducing the inflammatory response. These pathways offer promising therapeutic strategies for controlling severe and steroid-resistant asthma. NLRP3: Nod-like receptor protein 3, IL: Interleukin

NLRP3 has a role in pediatric asthma (especially non-allergic or steroid-resistant asthma) with increased serum levels along with IL-1β, and their level in induced sputum positively correlates with the severity of the disease [30]. MCC950, a highly selective NLRP3 inhibitor, and Ac-YVAD-cmk, a specific caspase-1 inhibitor, were experimented on murine models of neutrophilic (non-allergic) asthma induced by OVA combined with complete Freund’s adjuvant (CFA) and they significantly attenuated airway hyperresponsiveness, inflammation and reversed helper type 17, regulatory T cell imbalance in asthmatic mice suggesting a potential treatment to ameliorate airway inflammation [26]. MCC950 and Ac-YVAD-cmk are not yet approved as treatment for pediatric asthma, and they are still subjects of preclinical studies and animal models [67]. Another NLRP3 inhibitor is OLT1177 ® (dapansutrile) was experimented on OVA-induced asthma mouse, house dust mite induced asthma model and synthetic analogue of viral RNA Poly(I: C)-induced exacerbation of OVA asthma, and showed to reduce airway inflammation and lower eosinophils and Neutrophils counts in bronchoalveolar lavage fluid (BALF) in all models, in addition to finding comparable results in both oral and injections [68]. OLT1177 ® was experimented on humans for other diseases like congestive heart failure (CHF) and acute gout flares, and showed its efficacy and safety on humans [69], but none were conducted on asthmatic children. (Table 3, Fig. 4).

**Table 3:** Experimental NLRP3 Inhibitors and Their Therapeutic Potentials in Asthma

NLRP3 Inhibitor	Target	Effect in Asthma Models	Clinical Use Status	Reference
MCC950	NLRP3	Reduces airway inflammation	Preclinical	Chen et al., 2022
Ac-YVAD-cmk	Caspase-1	Reduces IL-1β, Th17 inflammation	Preclinical	Chen et al., 2022
OLT1177	NLRP3	Reduces eosinophil & neutrophil count	Studied in humans (not asthma)	Lunding et al., 2022

NLRP3: NOD-like receptor family pyrin domain-containing 3, Ac-YVAD-cmk: Acetyl-YVAD-chloromethylketone, IL: Interleukin, Th17: T helper 17 cells

### Downstream targeting

Asthma can have two major endotypes categories to be considered: T-helper type 2 cell high endotype (T2-High) which uses Interleukins 4 and 13 (IL-4, IL-13) causing production of immunoglobulin E (IgE) and IL-5 to bring eosinophils, T-helper type 2 cell low endotype (T2-Low) which uses IL-1, IL-8, IL-17, and Il-23 causing neutrophilic inflammation with normal serum and sputum eosinophils level [70]. Biologic agents such as omalizumab (anti-IgE), mepolizumab and benralizumab (anti-IL-5/IL-5R), and dupilumab (anti-IL-4Rα) are effective options for T2-high asthma, reducing exacerbations with good safety profiles. These therapies are approved for children and adults depending on age and eosinophil or FeNO levels. Anakinra, IL-1 receptor antagonist, is proposed to treat neutrophilic asthma and decreases levels of sputum neutrophils, IL-6, IL-8, and IL-1β. Canakinumab directly locks IL-1 with a longer half-life than Anakinra and has been proposed as to have potential therapeutic approach in neutrophilic asthma based on animal studies, mechanistic rationale, and preclinical models rather than human clinical trial evidence [71]. Anti-IL-1 therapies showed mixed results and outcomes in the context of obstructive airway diseases including asthma and COPD. According to some clinical studies, there were a modest reduction in airway inflammation, but others found no impact on overall disease progression [72]. Risankizumab, a humanized IgG1 monoclonal antibody that selectively targets IL-23, was proven to be ineffective and harmful in severe asthmatic adults [70]. Secukinumab, a human IgG1κ monoclonal antibody targeting IL-17A, is ineffective and would not bring clinical improvement in neutrophilic asthma [73]. Tezepelumab, a monoclonal antibody that targets thymic stromal lymphopoietin (TSLP) preventing interaction with its receptor thus blocks upstream activation of type 2 innate lymphoid cells (ILC2) finally blocking the release of IL-5, IL-4, IL-13, treats T-helper type 2 cell endotype High subtype asthma effectively and is approved for patients > 12 years with severe asthma regardless of serum eosinophils, IgE, and FeNO levels [74].

## Broader role of NLRP3 in pediatric inflammatory diseases

The NLRP3 inflammasome plays a central role in initiating the innate immune response by promoting the release of pro-inflammatory cytokines, particularly interleukin-1β (IL-1β) and interleukin-18 (IL-18), in response to PAMPs and DAMPs. These molecular signals act as alarms indicating the presence of pathogens or cellular damage. While this mechanism is essential for host defense and immune activation, its dysregulation can lead to pathological inflammation. Overactivation of NLRP3 has been implicated in several pediatric inflammatory disorders, including asthma, juvenile idiopathic arthritis (JIA), and inflammatory bowel disease (IBD), all of which share a common pathway involving NLRP3 activation and cytokine-driven inflammation. Given the central role of NLRP3 in the pathogenesis of these diseases and their overlapping mechanisms, there is growing interest in therapeutic strategies that target the NLRP3 inflammasome directly or modulate downstream cytokines such as IL-1β and IL-18. These approaches hold significant promise for managing coexisting inflammatory conditions and mitigating their long-term complications.

### Comparison with other diseases

The NLRP3 inflammasome plays a pivotal role in innate immunity by sensing danger signals such as pathogen‑associated and damage‑associated molecular patterns (PAMPs and DAMPs), ionic fluxes, and reactive oxygen species. Its activation triggers the maturation and release of IL‑1β and IL‑18, which drive inflammatory responses and, when uncontrolled, contribute to tissue damage [11, 23, 75]. While the primary focus of this review is asthma, it is important to note that dysregulated NLRP3 activity is also implicated in other chronic inflammatory diseases, particularly juvenile idiopathic arthritis (JIA) and inflammatory bowel disease (IBD) [7, 76, 77].

Despite their different initiating triggers, airway allergen exposure in asthma, microbial imbalance and dysbiosis in IBD, or genetic predisposition in JIA, these disorders converge on a common two‑signal model of NLRP3 activation. The priming step involves NF‑κB–mediated upregulation of NLRP3 and pro‑IL‑1β/IL‑18, followed by an activation phase leading to inflammasome assembly. This cascade culminates in the release of IL‑1β and IL‑18, promoting systemic and tissue‑specific inflammation [11, 75, 78]. In JIA, IL‑1β is recognized as a major driver of systemic inflammatory activity, while IL‑18 contributes to macrophage activation syndrome [79]. In IBD, NLRP3 activation has been linked not only to mucosal inflammation but also to intestinal fibroblast activation and fibrosis, contributing to stricture formation [76, 80].

These parallels highlight NLRP3 as a shared inflammatory pathway across diverse immune‑mediated diseases. In the context of pediatric asthma, this broader perspective underscores the importance of NLRP3 signaling as both a mechanistic contributor to airway inflammation and a potential biomarker of disease severity [81].

### Overlapping therapeutic strategies

Therapeutic strategies used in other NLRP3-related inflammatory diseases such as juvenile idiopathic arthritis and ulcerative colitis can also be applied to asthma. The activation of the NLRP3 inflammasome leads to the release of IL-1β, which then contributes to immune cell recruitment and tissue injury [75, 77]. Blocking IL-1β signaling with agents such as Anakinra, Canakinumab, and Rilonacept, which are already effective in the aforementioned diseases has shown significant improvement in controlling systemic inflammation [79, 83]. In addition, several NLRP3 inhibitors, including MCC950, OLT1177, Tranilast, CY-09, Oridonin, and RRx-001 are under investigation and have demonstrated the ability to suppress both inflammasome activation and downstream cytokine release [23, 76, 82].

## Clinical implications and future directions

The evidence discussed thus far demonstrates NLRP3’s role across multiple pediatric inflammatory diseases, including asthma. Translating results from both animal and human studies into clinical evidence raises several questions and highlights multiple issues that need to be addressed.

First, the use of NLRP3 markers in diagnosis and prognosis requires clear, standardized thresholds. A cross-sectional study by Li and Liu demonstrates the significantly elevated levels of serum NLRP3 in children with asthma in comparison to healthy controls. Their results demonstrated a positive correlation between sputum NLRP3 and IL-6, a negative correlation between sputum NLRP3 and FEV_1_/FVC, and an area under the curve of 0.758 [30]. This demonstrates the potential for NLRP3 in diagnosis and prognosis. Additional clinical studies are required to create standardized thresholds for serum and sputum NLRP3, as Li and Liu’s study was a single-center study with a limited sample size. Kim et al.’s animal study demonstrated that steroid-resistant asthma induced by Chlamydia and Haemophilus infections is characterized by elevated NLRP3 activity, IL-1β B levels, and neutrophilic airway inflammation [40]. In this model, elevated NLRP3 and IL-1 correlated with poorer asthma control and lower FEV1 values. The elevation of NLRP3 reflects the current lung function and aids in predicting clinical outcomes. Longitudinal studies are required to understand the relationship between NLRP3’s expression and hospitalization rate, steroid responsiveness, and exacerbation frequency. The integration of NLRP3 with other biomarkers could also enhance predictive accuracy.

Despite that, several limitations must be considered before integrating NLRP3 into clinical use. Biomarker interpretation can be confounded by other factors such as coexisting infections or genetic material. Additionally, if these biomarkers are integrated into diagnosis, it is important to note that there are no NLRP3 inhibitors approved for use in children, and only medications that block downstream inflammation mediated by NLRP3, like IL-1 inhibitors. Currently, no pediatric asthma guidelines include the use of inflammasome inhibition. The lack of approval for NLRP3 inhibitors is due to potential issues like toxicity and limited long-term data [84].

The lack of data and clinical trials regarding inflammasome inhibitors in the pediatric population is due to inherent challenges in this population. Trials involving young children face many problems, including consent and ethical concerns in regards to invasive sampling methods like induced sputum [85]. The existing data is cross-sectional, causing gaps in our understanding of how NLRP3 expression fluctuates within an individual during periods of disease exacerbation. The lack of longitudinal studies in the pediatric population represents a major limitation, as most existing studies focus on adults or use animal studies. Addressing these gaps will require a multi-center collaboration with minimally invasive diagnostic tools to further develop inflammasome targeting in pediatric asthma.

## Conclusion

The NLRP3 inflammasome has an important role in the pathogenesis of pediatric asthma, especially in neutrophilic corticosteroid-resistant types. Its main function is to amplify airway inflammation through the secretion of IL-1β and IL-18 cytokines, which bridge innate immune responses to chronic progression of the disease. Sputum-based trials showed a link between elevated NLRP3 expression and poor clinical outcomes, indicating that NLRP3 could be used as both a biomarker and a therapeutic target. The current treatments for pediatric asthma are corticosteroids and biologics, mainly primarily work by targeting type 2 inflammation and focus on symptom control. These approaches are less effective in the treatment of non-eosinophilic phenotypes.

New data from preclinical trials suggest that the use of NLRP3 inhibitors may be effective due to their role in reducing airway inflammation and maintaining immune balance. However, the application of these findings in clinical practice is challenging. Facing these challenges through conducting clinical trials and the development of new noninvasive diagnostic techniques may open new prospects for the diagnosis and treatment of pediatric asthma.

## Data Availability

Data sharing does not apply to this article as no datasets were generated or analysed during the current study.
